# Mice deficient in ER protein seipin have reduced adrenal cholesteryl ester lipid droplet formation and utilization

**DOI:** 10.1016/j.jlr.2022.100309

**Published:** 2022-11-01

**Authors:** Wen-Jun Shen, Yuan Cortez, Amar Singh, Weiqin Chen, Salman Azhar, Fredric B. Kraemer

**Affiliations:** 1 Division of Endocrinology, Gerontology, and Metabolism, Stanford University, Stanford, CA, USA; 2 Geriatric Research, Education, and Clinical Center, Veterans Affairs Palo Alto Health Care System, Palo Alto, CA, USA; 3 Department of Physiology, Augusta University, Augusta, GA, USA

**Keywords:** seipin deficiency, lipid droplet maturation, cholesterol, steroidogenesis, adrenal, testis, ovary, triacylglycerol, Berardinelli-Seip congenital lipodystrophy type 2, cholesterol trafficking, ACSL4, long-chain fatty acid coenzyme A synthetase 4, ACTH, adrenocorticotropic hormone, BSCL2, Berardinelli-Seip congenital lipodystrophy type 2, CE, cholesteryl ester, eCG, equine chorionic gonadotropin, ER, endoplasmic reticulum, hCG, human chorionic gonadotropin, h-HDL3, human HDL3, HSL, hormone-sensitive lipase, LD, lipid droplet, LDAF1, LD assembly factor 1, PMSG, pregnant mare's serum gonadotropin, SR-B1, scavenger receptor class B type 1, TAG, triacylglycerol, TMEM159, transmembrane 159

## Abstract

Cholesteryl ester (CE)-rich lipid droplets (LDs) accumulate in steroidogenic tissues under physiological conditions and constitute an important source of cholesterol as the precursor for the synthesis of all steroid hormones. The mechanisms specifically involved in CE-rich LD formation have not been directly studied and are assumed by most to occur in a fashion analogous to triacylglycerol-rich LDs. Seipin is an endoplasmic reticulum protein that forms oligomeric complexes at endoplasmic reticulum-LD contact sites, and seipin deficiency results in severe alterations in LD maturation and morphology as seen in Berardinelli-Seip congenital lipodystrophy type 2. While seipin is critical for triacylglycerol-rich LD formation, no studies have directly addressed whether seipin is important for CE-rich LD biogenesis. To address this issue, mice with deficient expression of seipin specifically in adrenal, testis, and ovary, steroidogenic tissues that accumulate CE-rich LDs under normal physiological conditions, were generated. We found that the steroidogenic-specific seipin-deficient mice displayed a marked reduction in LD and CE accumulation in the adrenals, demonstrating the pivotal role of seipin in CE-rich LD accumulation/formation. Moreover, the reduction in CE-rich LDs was associated with significant defects in adrenal and gonadal steroid hormone production that could not be completely reversed by addition of exogenous lipoprotein cholesterol. We conclude that seipin has a heretofore unappreciated role in intracellular cholesterol trafficking.

The mobilization of the cholesteryl esters (CEs) stored in CE-rich lipid droplets (LDs) appears to be the initially preferred source of cholesterol for steroidogenesis upon acute hormone stimulation. Evidence for this is based on data showing that ACSL4 (long-chain fatty acid coenzyme A synthetase 4) deficiency in steroidogenic tissues results in markedly reduced adrenal CE stores that are associated with defective steroidogenesis in vivo, which is exacerbated ex vivo and in vitro when exogenous lipoproteins are unavailable, meaning conditions in which LDs, plasma membrane cholesterol, and cholesterol synthesis are the primary sources of cholesterol precursor for steroidogenesis (1). The fact that steroidogenesis is restored to normal when lipoproteins are supplied as a source of cholesterol (2) establishes the importance of LD cholesterol as the initial preferred source for steroid production. In steroidogenic tissues, CE-rich LDs can accumulate either through the uptake of intact CEs from circulating lipoproteins mediated by scavenger receptor class B type 1 (SR-B1) or through the esterification of unesterified cholesterol, derived from either de novo synthesis or receptor-mediated endocytosis of lipoproteins, via the actions of ACAT within the endoplasmic reticulum (ER). The mechanisms specifically involved in CE-rich LD formation have not been directly studied and are assumed by most to occur in a fashion analogous to triacylglycerol (TAG)-rich LDs (3); however, it is apparent that CE-rich and TAG-rich LDs have important differences beyond their lipid compositions since it has been shown that the proteomes of CE-rich and TAG-rich LDs display significant differences (4). Further evidence to support different mechanisms for formation of CE-rich and TAG-rich LDs is derived from patients with generalized lipodystrophy who have markedly reduced ability to form TAG LDs, but who, nonetheless, develop atherosclerosis with its characteristic CE-LD deposits in lesions (5). Unesterified cholesterol is produced by the hydrolysis of CEs within cytosolic LDs via hormone-sensitive lipase (HSL) (6, 7, 8). Interestingly, the CEs in LDs undergo continuous hydrolysis and re-esterification (9). Although it has been suggested that several lipid synthetic enzymes are components of the LD proteome (10) and that lipid synthesis can take place on the LD itself, recent observations suggest that all cytosolic LDs, no matter their subcellular location, are continuously and intimately in contact with the ER (11). This means that LD turnover leads to LD-cholesterol continuously moving into the ER, either to be trafficked to other organelles in the cell or to be re-esterified back into LDs.

The most common and severe form of generalized lipodystrophy is seen in Berardinelli-Seip congenital lipodystrophy type 2 (BSCL2), where mutations in BSCL2 (also named seipin) are responsible (12). Seipin is an ER protein that forms oligomeric complexes at ER-LD contact sites and whose deficiency results in severe alterations in LD maturation and morphology (13). Seipin reportedly interacts with a number of ER protein partners, including with TMEM159 (transmembrane 159), also known as promethin (14) and renamed LDAF1 (LD assembly factor 1), to form a large oligomeric complex that facilitates and determines the site of LD biogenesis in the ER (15). Both seipin and LDAF1 are ubiquitously expressed in tissues, but no studies have directly addressed whether they are important for CE-rich LD biogenesis.

## Materials and methods

### Chemicals and reagents

Reagents were obtained from the following sources: Cholesterol LiquiColor Test (enzymatic) from Stanbio (Boerne, TX); bicinchoninic acid assay protein kit from Pierce Biotechnology, Inc (Rockford, IL); organic solvents were from J. T. Baker (Phillipsburg, NJ); TRIzol reagent and SuperScript II were from Invitrogen (Carlsbad, CA); RNeasy kit from QIAGEN (Valencia, CA); SYBR Green TaqMan PCR kit from Applied Biosystems (Foster City, CA); corticosterone ELISA kit from Cayman Chemical (Ann Arbor, MI); progesterone and testosterone ELISA kits from ALPCO (Salem, NH); pregnant mare's serum gonadotropin (PMSG)/equine chorionic gonadotropin (eCG), human chorionic gonadotropin (hCG), and Bt_2_cAMP from MilliporeSigma (St. Louis, MO). Cortrosyn was from Amphastar Pharmaceuticals, Inc (Rancho Cucamonga, CA).

### Animals

Animal studies were performed in accordance with the National Institutes of Health guidelines, and all procedures were approved by the institutional animal care and use committee of VA Palo Alto Health Care System. *Bscl2*^*f/f*^ mice on a C57BL/6J background in which exon 3 of mouse *Bscl2* gene and its flanking intronic region was flanked by two loxP sites were generated as previously described (16). Deletion of exon 3 not only removes a portion of the coding gene but also leads to a frameshift mutation generating a premature stop codon one amino acid after the presumed exon skipping (16). The animals carrying the floxed *Bscl2* were crossed with transgenic mice expressing Cre recombinase under control of the *Cyp11a1* promoter on a C57BL/6J background, obtained from Jackson Laboratories (stock number: 010988). Cre activity is expressed in the adrenals of both sexes and in the testes of these animals leading to the knockdown of seipin in the adrenals and testes. All animals were maintained on a chow diet (TD 2918) with a 12 h/12 h dark-light cycle. Genotypes were identified by PCR analysis using genomic DNA from tail biopsies as described. Both male and female mice were studied and analyzed separately for gender-specific differences in responses. *Bscl2* floxed mice and CYP11A1-Cre mice were analyzed separately as control animals. *Bscl2* KO and control mice aged 16–24 weeks were used for the experiments. To assess serum corticosterone production, control and *Bscl2* KO mice were injected with adrenocorticotropic hormone (ACTH) (Cortrosyn, 2.5 IU/mouse) subcutaneously once. Blood was drawn from mice prior to and 1 h after ACTH injection. To assess serum testosterone production, male animals were treated with hCG (2.5 IU/mouse), and serum was collected 4 h later. To assess progesterone production, female mice were treated with 5 IU of PMSG/eCG, and serum was collected 5 h later.

For detection of *Bscl2* mRNA expression in different tissues, mice were injected with saline or ACTH subcutaneously once, and various tissues were collected 1 h postinjection, frozen in liquid nitrogen, and stored at −80°C for later use.

### Ex vivo adrenal steroid production

*Bscl2* KO and control mice were sacrificed at 24 weeks old, and the adrenals were removed. Adrenals were cut into half, and each half adrenal was preincubated in one tube of 500 μl medium (RPMI1640 and supplemented with 10% FBS) at 37°C 5% CO_2_ for 1 h, then transferred to another tube, and treated in 500 μl medium with 2.5 mM Bt_2_cAMP at 37°C 5% CO_2_ for another 1 h. Some experiments included the presence or absence of human HDL3 (h-HDL_3_; 500 μg cholesterol/ml) in the incubation. HDL_3_ (d: 1.125 and 1.210 g/ml) was isolated from fresh plasma of healthy male donors as previously described (17). Media were collected after incubation and subjected to ELISA analysis of corticosterone.

### Preparation and treatment of granulosa and Leydig cells

Granulosa cells from control and *Bscl2* KO mice were prepared as described previously (18, 19). Briefly, immature female mice (22–25 days old) were injected once with 5 IU of PMSG/eCG for 48 h. After hormone treatment, the ovaries were excised and placed in basal medium (DMEM:F12 with 20 mM Hepes [pH 7.4]) supplemented with 100 U/ml penicillin and 100 μg/ml streptomycin. Clumps of mural granulosa cells and oocyte-cumulus complexes were released into the medium by puncturing follicles with a 25-gauge needle. The mural granulosa cells were collected in DMEM/F12/Hepes/BSA medium and dispersed by being gently drawn in and out of a Pasteur pipette. The granulosa cells were washed and resuspended in fresh medium and cultured as described previously (20, 21, 22). The cells were then incubated with Bt_2_cAMP in the presence or absence of h-HDL_3_ (500 μg cholesterol/ml) for 5 h in triplicate. After the incubation, media were collected, and progesterone production was measured using ELISA.

For isolation of Leydig cells, testicular interstitial cells containing Leydig cells were isolated by mechanical dispersion of decapsulated testis obtained from control and *Bscl2* KO mice. Highly purified (>85%) Leydig cell preparations were obtained by subjecting interstitial cell suspensions to Percoll density gradient centrifugation as previously described (19, 23). To assay steroidogenesis, freshly purified Leydig cells were incubated with Bt_2_cAMP (2.5 mM) in the presence or the absence of h-HDL_3_ (500 μg cholesterol/ml) for 5 h in triplicate, and secreted testosterone was assayed by ELISA (24).

### RNA isolation and quantitative real-time PCR analysis

For RNA isolation, tissues were homogenized in 1 ml of TRIzol reagent using a power homogenizer (Ultra-Turrax T25; Labortechnik, Gottingen, Germany). RNA was isolated following the protocol for TRIzol reagent, and after the isopropanol precipitation step, total RNA was dissolved in 20 μl RNase-free water, RNA concentration and quality were analyzed, and then 1 μg RNA were converted to complementary DNA using SuperScript II reverse transcriptase (Invitrogen) for real-time PCR analysis. Real-time PCR was performed with the complementary DNA prepared as above using an ABI Prism 8500 System using SYBR Green master mix reagent. The relative mass of specific RNA was calculated by the comparative cycle of threshold detection method according to the manufacturer's instruction. The following were the genes examined: Plin1, Plin2, HSL, CGI-58, SR-B1, LRP1, LDLR, Aster B, TMEM159, CYP11A1, CYP11B1, CYP21A1, 3ß-HSD, StAR, SNAP23, SNAP25, and αSNAP. Supplemental Table S1 shows the primer sets used for each gene.

### Lipid extraction

Lipid extraction from adrenals was performed as described previously (25). Briefly, adrenal tissue extract was mixed with 20 times volume of chloroform/methanol (2:1) and vortexed. Following that, chloroform was added to the mixture and subjected to centrifugation. The combined lower layers were washed and transferred to a new tube and dried under nitrogen. The lipids were dissolved with 20 μl ethanol. Total cholesterol and free cholesterol were measured using LiquiColor Test (enzymatic) kits.

### Immunocytochemistry and Oil Red O staining

Immunocytochemistry was performed as previously described (2). Briefly, adrenal, ovary, and testis tissues were fixed in 10% formalin overnight before processed and embedded in paraffin for section. Slides were sectioned at 10 μm and stained with anti-Bscl2 (1:200 dilution; Thermo Fisher Scientific, Waltham, MA) or mouse IgG as negative control. For Oil Red O staining, adrenals were fixed in Tissue-Tek optimal cutting temperature (Sakura Finetek USA, Torrance, CA) and processed into 10 μm sections before staining with Oil Red O. Images were quantified using ImageJ software from the National Institutes of Health.

### Electron microscopy

Adrenals from control and seipin KO mice were processed for electron microscopy by standard techniques in the Stanford University Cell Sciences Imaging Core Facility. In brief, adrenals were fixed in Karnovsky’s fixative: 2% glutaraldehyde (EMS; catalog no.: 16000) and 4% paraformaldehyde (EMS; catalog no.: 15700) in 0.1 M sodium cacodylate (EMS; catalog no.: 12300) pH 7.4 for 1 h. The fix was replaced with cold/aqueous 1% osmium tetroxide (EMS; catalog no.: 19100) and were then allowed to warm to room temperature for 2 h rotating in a hood, washed 3× with ultrafiltered water, then en bloc stained in 1% uranyl acetate at room temperature for 2 h while rotating. Samples were then dehydrated in a series of ethanol washes for 30 min each at room temperature beginning at 50%, 70% EtOH then moved to 4°C overnight. They were placed in cold 95% EtOH and allowed to warm to room temperature, changed to 100% 2×, then propylene oxide for 15 min. Samples were infiltrated with EMbed-812 resin (EMS; catalog no.: 14120) mixed 1:2, 1:1, and 2:1 with propylene oxide for 2 h each with leaving samples in 2:1 resin to propylene oxide overnight rotating at room temperature in the hood. The samples were then placed into EMbed-812 for 2–4 h, then placed into molds with labels and fresh resin, orientated, and placed into a 65°C oven overnight. Sections were taken around 80 nm using an UC7 (Leica, Wetzlar, Germany) picked up on formvar/carbon-coated 100 mesh Cu grids, stained for 40 s in 3.5% uranyl acetate in 50% acetone followed by staining in Sato’s Lead Citrate for 2 min, then observed in the JEOL JEM-1400 120 kV. Images were taken using a Gatan Orius 832 4k X 2.6k digital camera with 9 μm pixel. Images were quantified using ImageJ software. Briefly, for quantification of LD size, LDs were surrounded with a perimeter, and the area was measured. Three pictures of 23.4 × 23.4 in with 3,000× magnification and three pictures of 23.4 × 23.4 in with 6,000× magnification were chosen randomly and analyzed for male control and seipin KO adrenal samples.

### Statistics

Data are expressed as means ± SEM. Statistical analyses were performed by one-way ANOVA and independent *t*-test using SPSS 20.0 and GraphPad Prism 8.0 (La Jolla, CA). Differences between groups were considered statistically significant when *P* < 0.05.

## Results

### Generation of steroidogenic tissue-specific seipin KO mice

**Fig. 1:**
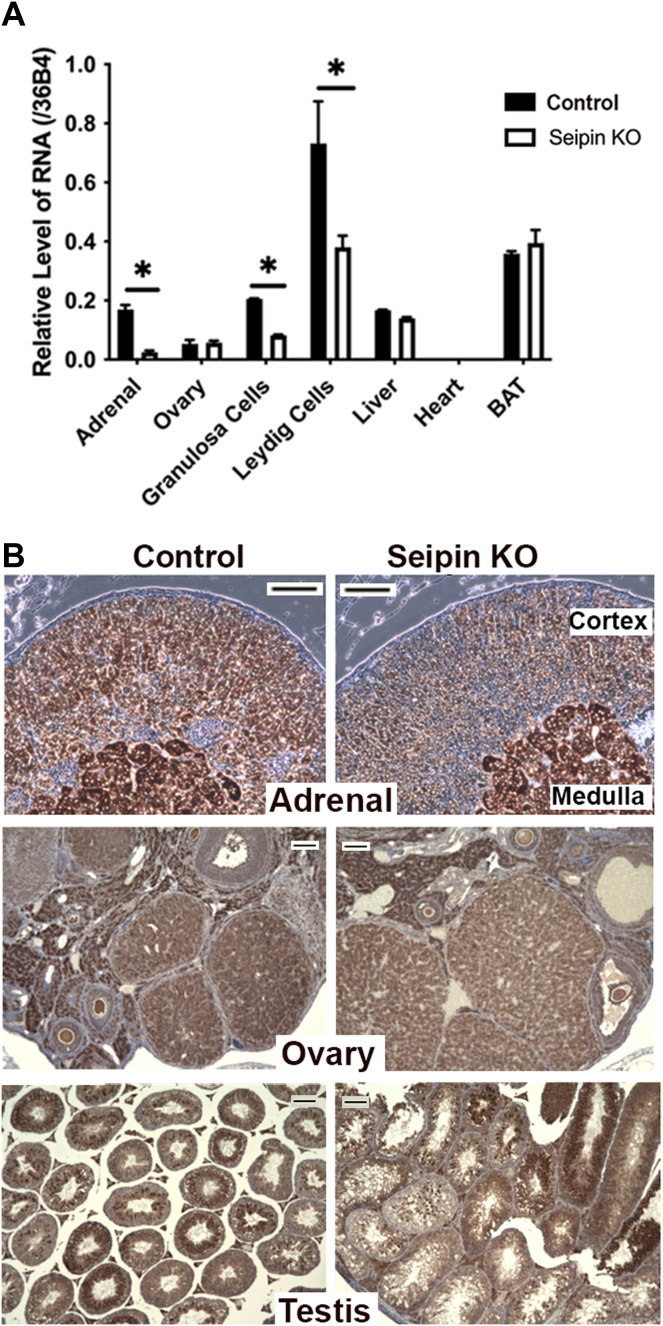
Generation of steroidogenic tissue-specific seipin KO mice. A: Bcsl2 (seipin) mRNA expression in tissues of control and seipin KO mice. mRNA was isolated from adrenal and other tissues (n = 4) and analyzed using TaqMan quantitative PCR as described in the Materials and methods section. Data are expressed as mean ± SEM. B: Immunohistochemical staining of seipin in adrenal, testis, and ovary of control and seipin KO mice (scale bar in each panel represents 100 μm).

To generate steroidogenic tissue-specific KO of seipin mice, animals carrying the floxed *Bscl2* (16) were crossed with CYP11A1-Cre mice, where Cre expression is primarily restricted to steroid-producing cells. After confirmation of genotype by PCR from tail genomic DNA, the expression of Bscl2 mRNA in the adrenal and other tissues was analyzed using TaqMan quantitative PCR. As shown in Fig. 1A, the expression of Bscl2 mRNA was reduced ∼80% in the adrenal (*P* < 0.01), ∼50% in Leydig cells (*P* < 0.05), and ∼40% in granulosa cells (*P* < 0.05) of KO mice compared with control, whereas Bscl2 mRNA expression was unchanged in ovary, heart, liver, or brown adipose tissue. Further analysis of protein levels using anti-Bscl2 antibody for histochemical staining of tissue sections from adrenals (Fig. 1B) showed distinct staining of seipin in the adrenal cortex of control mice, but these signals were significantly reduced in adrenal sections from seipin KO mice. Seipin staining was detected in the adrenal medulla, since CYP11A1 is not expressed in the adrenal medulla. Seipin staining in testis and ovary of seipin KO mice was comparable to control, owing to the expression of CYP11A1-Cre in a minority number of cells in these tissues.

**Fig. 2:**
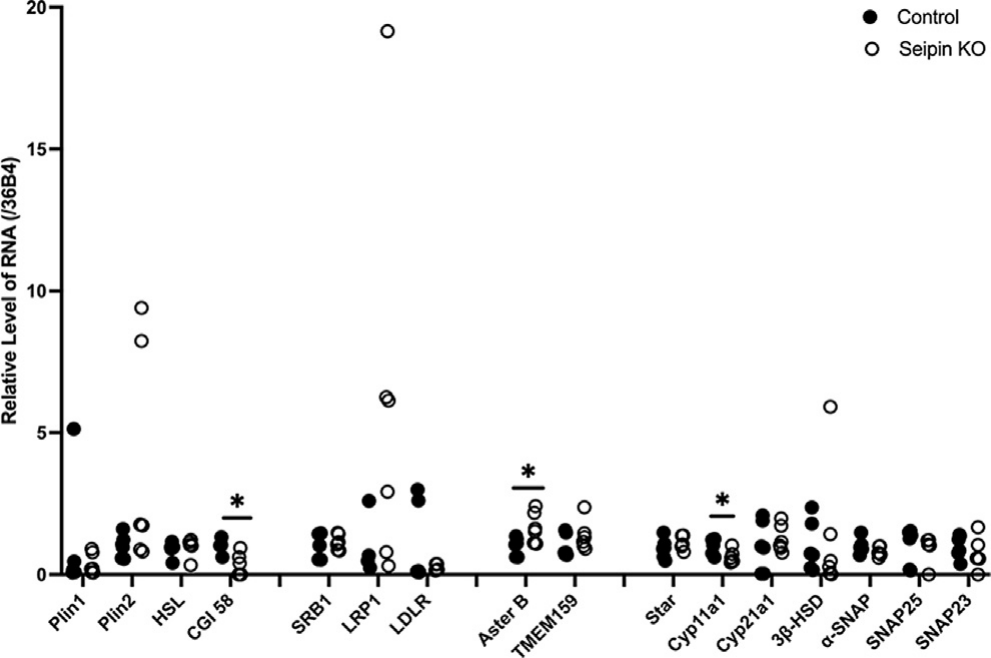
Analysis of expression of steroidogenic-related genes in adrenals. mRNA was isolated from the adrenals of control (n = 4) and seipin KO mice (n = 4) and the expression of various genes analyzed using TaqMan quantitative PCR as described in the Materials and methods section. Data are displayed. ∗*P* < 0.05.

To further analyze the effect of seipin ablation, the expression of some select genes that are involved in LD metabolism and steroidogenesis was analyzed using TaqMan quantitative PCR. As shown in Fig. 2, the mRNA levels of most of the genes analyzed, Plin1, Plin2, HSL, SR-B1, LRP1, LDLR, TMEM159, Star, Cyp21a1, 3ßHSD, α-SNAP, SNAP25, and SNAP23, showed no differences in the adrenals between control and seipin KO mice. The mRNA levels of CGI-58 and Cyp11a1, however, appeared to be decreased, whereas Aster B mRNA was increased in the adrenals of seipin KO mice.

### Adrenal LDs and lipids in control and seipin KO mice

**Fig. 3:**
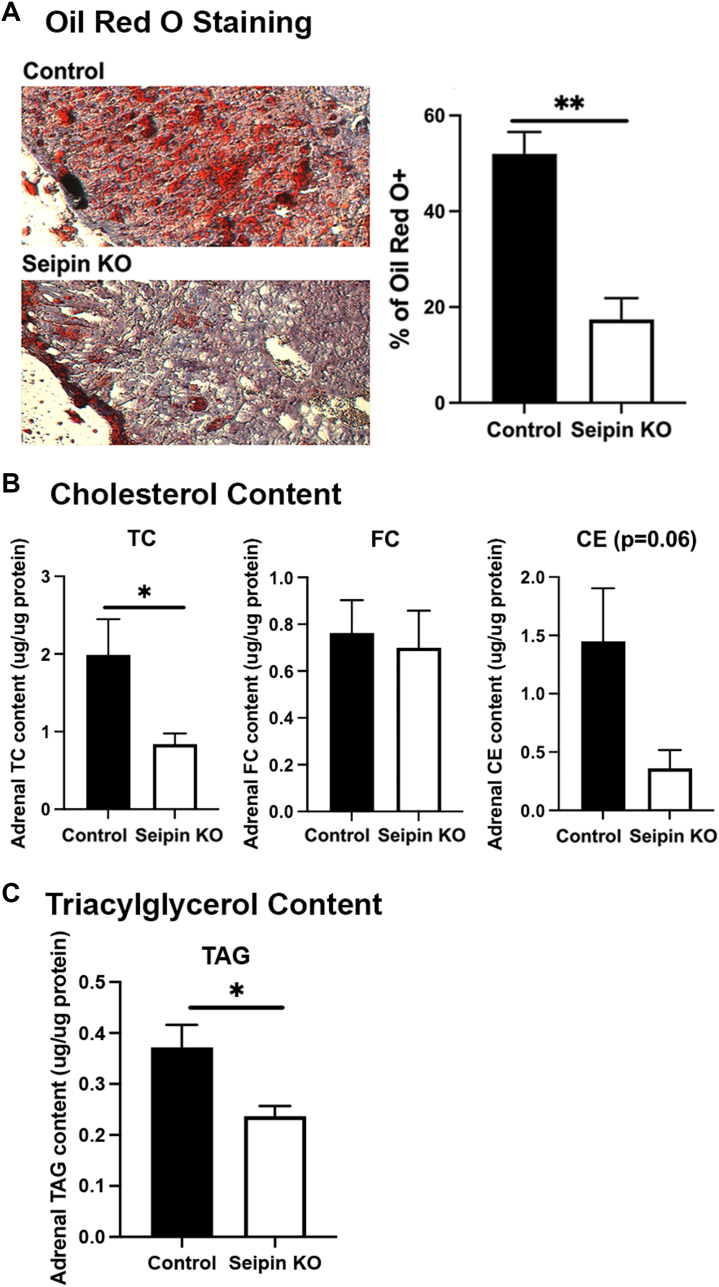
Adrenal LDs. A: Oil Red O staining of adrenals in control (n = 4) and seipin KO mice (n = 4). Scale bars represent 100 μm. B: Total cholesterol (TC), free unesterified cholesterol (FC), and CE contents in control and seipin KO mice adrenals. C: TAG content in control and seipin KO mice adrenals. Total lipids were extracted from the adrenals of control (n = 7) and seipin KO male mice (n = 7) using the Folch method. Total cholesterol, unesterified cholesterol, CEs, and TAG were assayed as described in the Materials and methods section. Data are expressed as mean ± SEM. ∗*P* < 0.05.

Since seipin was successfully knocked down, but not completely eliminated, in the adrenal, the effects of seipin deficiency on adrenal lipid content were examined. Oil Red O staining of adrenal sections (Fig. 3A) revealed a marked >60% reduction in LDs in adrenals of seipin KO mice compared with floxed controls, consistent with seipin being necessary for the accumulation of CE-rich LDs. Measurement of cholesterol content documented that total cholesterol content was reduced ∼50% (*P* < 0.05), reflecting a reduction in CE content of ∼70% (*P* = 0.06) in seipin KO adrenals (Fig. 3B), whereas free cholesterol content was similar in adrenals of seipin KO and floxed control mice. Adrenal TAG content was also reduced ∼30% in seipin KO compared with floxed control mice (Fig. 3C).

**Fig. 4:**
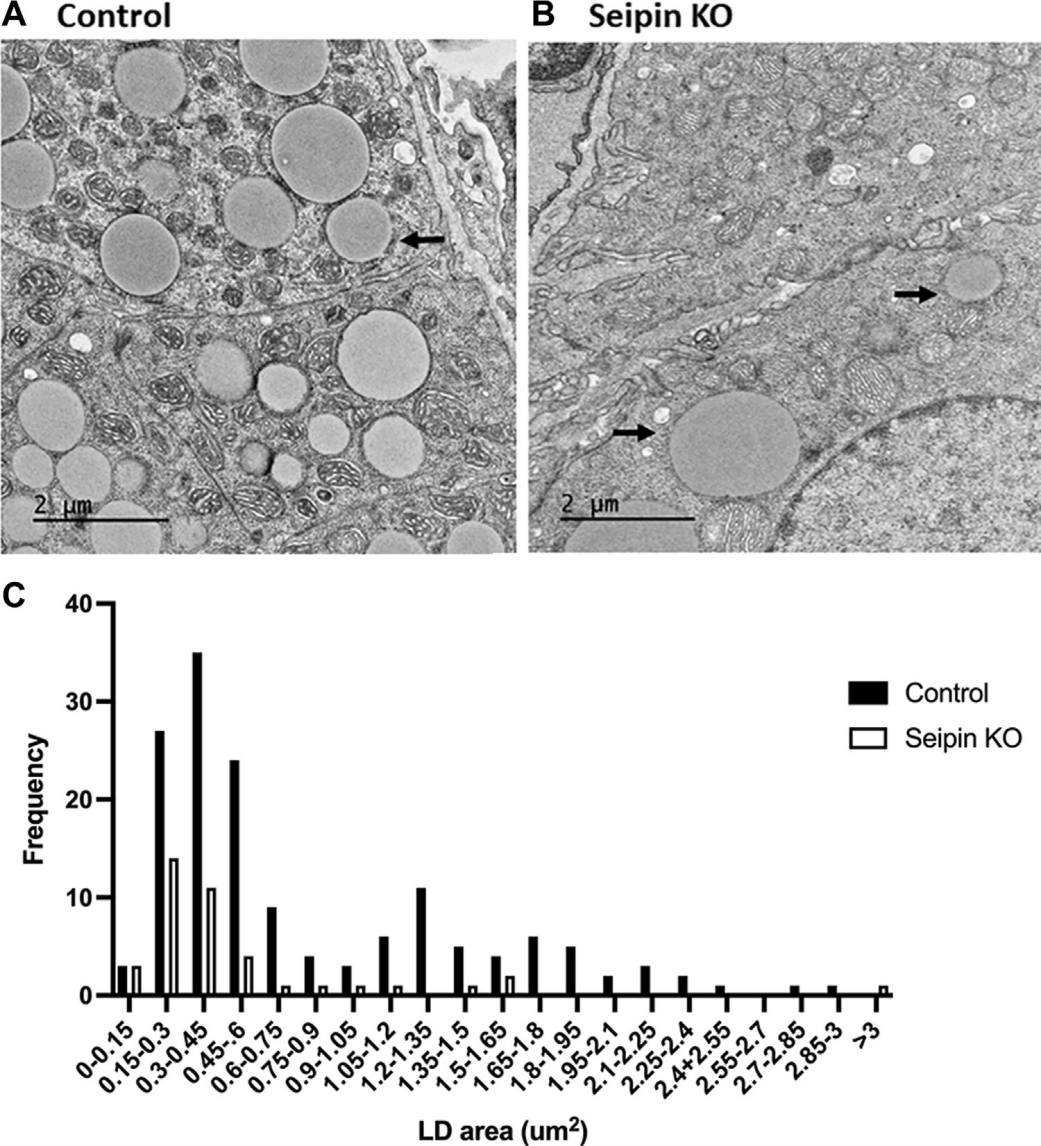
Electron microscopy of adrenal LDs. A: Control. Magnification 3,000×. Arrow denotes LDs. B: Seipin KO. Magnification 3,000×. Arrow denotes LDs. C: Frequency distribution of the size of LDs determined using ImageJ software.

To further examine the effects of seipin deficiency on adrenal LDs, electron microscopy analysis of adrenal sections was performed (Fig. 4). As shown in Fig. 4A, there were numerous LDs observed in sections of adrenals from control mice. In contrast, LDs were sparse in sections of adrenals from seipin KO mice (Fig. 4B). When the number and size distribution of LDs were quantified using ImageJ software (Fig. 4C), there was a 75% reduction in the number of LDs observed in adrenals from seipin KO compared with control mice, but the size distribution of the LDs was similar in seipin KO and control mice. It is interesting that the 75% reduction in LDs in seipin KO mice roughly parallels the ∼80% reduction of seipin mRNA in these animals, suggesting that the presence of LDs in the seipin KO adrenals is due to residual seipin expression and the relative efficiency of the cre recombinase.

### Effects of tissue-specific ablation of seipin on stimulated adrenal steroids

**Fig. 5:**
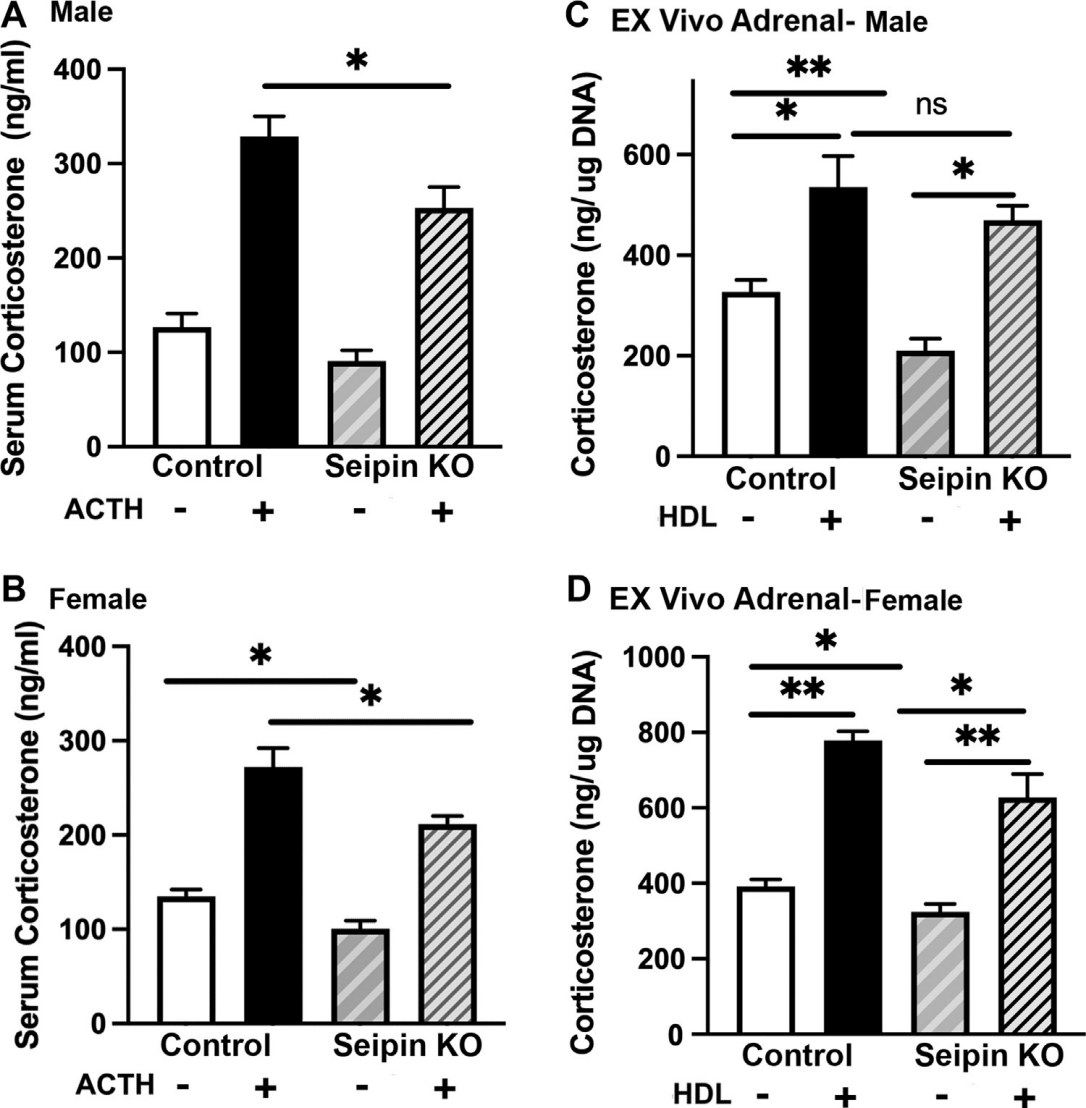
Corticosterone production in control and seipin KO mice. A: Corticosterone production in control and seipin KO male mice. Male control (n = 8) and seipin KO mice (n = 8) were treated with cosyntropin (2.5 IU per mouse, 1 h) and serum collected before and 1 h after the treatment. B: Corticosterone production in control and seipin KO female mice. Female control (n = 8) and seipin KO mice (n = 8) were treated with cosyntropin (2.5 IU per mouse, 1 h) and serum collected before and 1 h after the treatment. C: Ex vivo corticosterone production in male control and seipin KO mice (n = 8). Adrenals from male mice were cut in half and cultured ex vivo. After 0.5 h, the media were changed with fresh media with 2.5 mM Bt_2_cAMP in the absence or presence of h-HDL_3_ (500 μg cholesterol/ml). After 1 h incubation, corticosterone levels in the media were measured. D: Ex vivo corticosterone production in female control (n = 8) and seipin KO mice (n = 8). Adrenals from female mice were cut in half and cultured ex vivo. After 0.5 h, the media were changed with fresh media with 2.5 mM Bt_2_cAMP in the absence or presence of h-HDL_3_ (500 μg cholesterol/ml). After 1 h incubation, corticosterone levels in the media were measured. Data are expressed as mean ± SEM. ∗*P* < 0.05; ∗∗*P* < 0.01.

To analyze the role of seipin in adrenal steroidogenesis, both male and female control and seipin KO mice were treated with cosyntropin and serum collected 1 h later for analysis of corticosterone levels. As shown in the male mice (Fig. 5A), compared with control floxed mice, maximal corticosterone levels were reduced 23% in male seipin KO mice (*P* < 0.05). In the female mice, both basal (26%, *P* < 0.05) and maximum levels of corticosterone after ACTH stimulation (22%, *P* < 0.05) were statistically significantly reduced in seipin KO mice compared with control (Fig. 5B).

### Decreased steroidogenesis ex vivo in adrenals from seipin KO mice

To further explore potential changes in circulating serum corticosterone, adrenals from male and female control and seipin KO mice were dissected and placed in culture medium after dissecting in half. Corticosterone levels in the media were measured after treatment with 2.5 mM Bt_2_cAMP in the presence or absence of h-HDL_3_ for 1 h. As shown in Fig. 5C, D, similar to the in vivo observation, corticosterone levels in response to cAMP treatment in the absence of HDL were significantly lower in the adrenals from seipin KO mice compared with control in both male (*P* < 0.01) and female (*P* < 0.05) mice. Incubation of adrenals with cAMP in the presence of HDL significantly increased corticosterone production in both control and seipin KO mice, with the inclusion of HDL increasing corticosterone production in adrenals of male seipin KO mice that was not significantly different from control; however, the presence of HDL, while increasing corticosterone production in female seipin KO mice adrenals, failed to increase corticosterone production to the same degree as in female control adrenals. These results are consistent with most or all the reduction in ex vivo production of corticosterone in seipin KO adrenals being because of reduced cellular CE stores, as previously demonstrated in ACSL4 KO mice (2).

### Effects of tissue-specific ablation of seipin on stimulated gonadal steroids

**Fig. 6:**
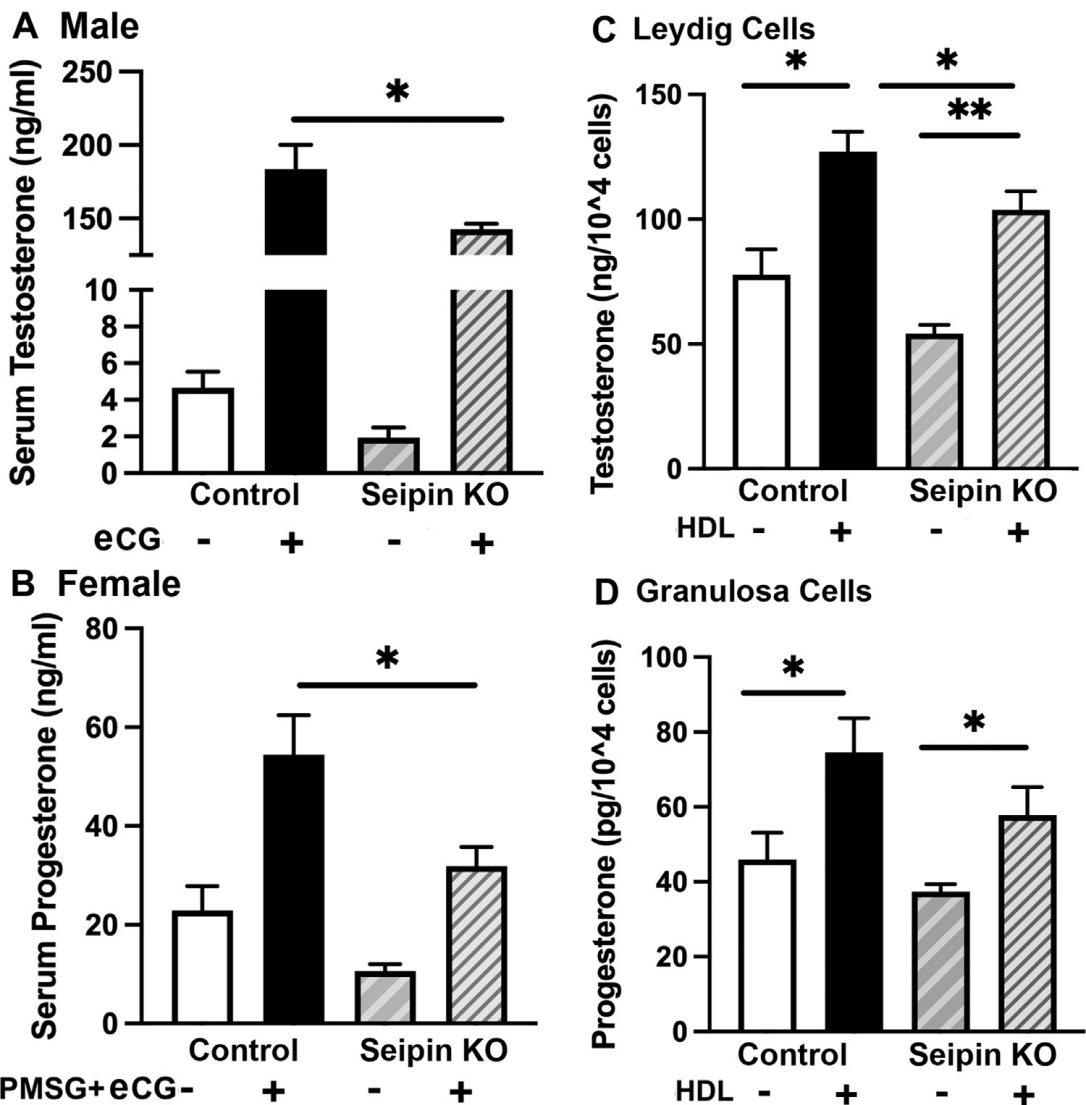
Gonadal steroid production in control and seipin KO mice. A: Serum testosterone in control and seipin KO male mice. Male control (n = 8) and seipin KO mice (n = 8) were treated with hCG (2.5 IU per mouse), and serum was collected before and 4 h after the treatment for measurement of testosterone. B: Progesterone production in control and seipin KO female mice. Female control (n = 8) and seipin KO mice (n = 8) were treated with PMSG/eCG (2.5 IU per mouse), and serum was collected before and 5 h after the treatment for measurement of progesterone. C: Testosterone production in isolated Leydig cells. Leydig cells were isolated from control (n = 8) and seipin KO mice (n = 8) using Percoll gradients as described in the Materials and methods section. Freshly purified Leydig cells were incubated with 2.5 mM Bt_2_cAMP in the absence or presence of h-HDL_3_ (500 μg cholesterol/ml). After 5 h incubation, testosterone levels in the media were measured. D: Progesterone production in isolated granulosa cells. Age-matched female control (n = 8) and seipin KO mice (n = 8) were injected twice with 5 IU PMSG for 48 h. Granulosa cells were then isolated as described in the Materials and methods section. Freshly purified granulosa cells were incubated with 2.5 mM Bt_2_cAMP in the absence or presence of h-HDL_3_ (500 μg cholesterol/ml). After 5 h incubation, progesterone levels in the media were measured. Data are expressed as mean ± SEM. ∗*P* < 0.05; ∗∗*P* < 0.01.

To examine the role of seipin in gonadal steroidogenesis, male control and seipin KO mice were treated with saline or hCG, and serum was collected 4 h later for analysis of testosterone levels (Fig. 6A). After hCG treatment, testosterone levels were reduced ∼22% in male seipin KO mice (*P* < 0.05). To examine the role of seipin in ovarian steroidogenesis, female control and seipin KO mice were treated with PMSG/eCG, and serum was collected 4 h later for analysis of progesterone levels (Fig. 6B). After PMSG/eCG treatment, progesterone levels were significantly reduced in female seipin KO mice, to only ∼58.7% of that of female control mice (*P* < 0.05).

### Decreased steroidogenesis ex vivo in Leydig and granulosa cells from seipin KO mice

To further explore potential changes in circulating serum gonadal steroids, Leydig cells were isolated from male testes and granulosa cells were isolated from female ovaries of control and seipin KO mice. Testosterone (Leydig cells) and progesterone (granulosa cells) levels in the media were measured after treatment with 2.5 mM Bt_2_cAMP in the presence or absence of h-HDL_3_ for 1 h. As shown in Fig. 6C, incubation of Leydig cells with cAMP in the presence of HDL significantly increased tesosterone production in both control and seipin KO mice, but the inclusion of HDL failed to normalize testosterone production in seipin KO mice. As shown in Fig. 6D, incubation of granulosa cells with cAMP in the presence of HDL significantly increased progesterone production in both control and seipin KO mice, but the inclusion of HDL also failed to normalize progesterone production in seipin KO mice. It is noteworthy that the fold increase in steroid hormone production in response to the addition of HDL in the control and seipin KO gonadal cells is the same, although the basal and final stimulated steroid production in the seipin KO gonadal cells are less than that of the control gonadal cells.

## Discussion

The current studies were undertaken to assess whether seipin deficiency influences CE-rich LD accumulation in steroidogenic tissues, particularly the adrenal, and to determine whether any alterations in LD accumulation affect steroid hormone production. To address these questions, we generated steroidogenic tissue-specific KO of seipin by crossing animals carrying the floxed *Bscl2* with CYP11A1-Cre mice, where Cre expression is primarily restricted to steroid-producing cells. It is notable that global seipin KO male mice have been reported to be infertile, owing to an important role of seipin in germ cells and spermatogenesis (26). Although we did not attempt to mate steroidogenic tissue-specific seipin KO mice in the current studies, an effect on male fertility might not be expected since KO of seipin was restricted to Leydig cells, where CYP11A1 is expressed (27), sparing germ cells and sperm, and testosterone levels, though significantly reduced, were only moderately affected. After documenting the successful knockdown of seipin in adrenals, Leydig, and granulosa cells of the mice, we observed a dramatic reduction in lipid accumulation (Oil Red O staining) in the adrenals of seipin KO mice, which was confirmed to reflect a reduction in total cholesterol and CE content, without a change in free (unesterified) cholesterol content, in addition to a reduction in cellular TAG content. Moreover, there was a 75% reduction in the number of LDs observed by electron microscopy in adrenals from seipin KO without a change in the size distribution of the LDs. It is interesting that the 75% reduction in LDs in seipin KO mice roughly parallels the ∼80% reduction of seipin mRNA in these animals, suggesting that the presence of LDs in the seipin KO adrenals is due to residual seipin expression. Thus, seipin appears to be critical for the accumulation and, presumably formation, of CE-rich LDs in the adrenal. Seipin is an ER protein that forms oligomeric complexes at ER-LD contact sites and whose deficiency results in severe alterations in TAG-rich LD maturation and morphology (13). Seipin reportedly interacts with a number of ER protein partners, including with TMEM159, also known as promethin (14) and renamed LDAF1, to form a large oligomeric complex that facilitates and determines the site of LD biogenesis in the ER (15). It is noteworthy that knockdown of seipin had no impact on the expression of TMEM159 mRNA in the adrenal, but we acknowledge that protein levels of TMEM159 were not assessed. The observation that seipin deficiency resulted in a marked reduction in CE-rich LDs raises mechanistic questions, which have not been addressed in the current studies. For example, since CE-rich LDs also contain TAG (4, 28, 29), does seipin contribute to CE-rich LD accumulation via the formation of a TAG nidus that supports subsequent incorporation of CEs into the LD? Alternatively, does the seipin-TMEM159 complex channel CEs formed in the ER via the esterification of cholesterol by the actions of ACAT directly into LDs? These issues will require further experimental approaches in the future.

In view of the reduced accumulation of CE-rich LDs in the adrenals and presumably in Leydig and granulosa cells (even though lipid composition was not directly assessed in these cells in the current studies) in seipin KO mice, it is not surprising that seipin KO mice displayed a reduced capacity to produce steroid hormones, corticosterone, testosterone, and progesterone, when stimulated by ACTH, hCG, and PMSG/eCG, respectively, since stored CEs are an essential initial source of cholesterol for steroidogenesis (2, 30). This defect in steroidogenesis is more remarkable when considered in the context that the knockdown of seipin was incomplete, achieving an ∼80% reduction in adrenocortical cells, ∼50% reduction in Leydig cells, and ∼40% reduction in granulosa cells. It is interesting that the ex vivo incubation of adrenals, Leydig cells, and granulosa cells with cAMP and HDL failed to normalize steroid hormone production. This contrasts with the previous observations in steroidogenic tissue-specific ACSL4 KO mice where the defects in steroid production in adrenals, Leydig cells, and granulosa cells because of reduced CE accumulation were corrected by incubation with HDL (2). Assessment of the expression of a variety of genes involved in steroidogenesis did not reveal any substantial alterations that could explain this result; however, we acknowledge that gene expression was only examined in adrenals and not in either Leydig or granulosa cells, leaving open the possibility that knockdown of seipin could have resulted in changes in some steroidogenic genes in these tissues, though unlikely. The inability of exogenous lipoprotein cholesterol to normalize steroid production in seipin KO mice, particularly in Leydig and granulosa cells, raises the possibility that knockdown of seipin could have impaired the trafficking of cholesterol derived from HDL. CEs contained within HDL are selectively taken up intact into cells via SR-B1 and then hydrolyzed by HSL within the cytosol (1, 31). The released unesterified cholesterol is re-esterified within the ER by ACAT. In view of the pivotal role of the ER in cholesterol trafficking (32), it is possible that knockdown of seipin could somehow have impaired the trafficking of cholesterol from the ER to mitochondria for steroidogenesis either by altering the architecture of the ER and/or the orientation or function of ER-intrinsic proteins that are involved in cholesterol trafficking. These possibilities will need to be addressed in future studies.

In summary, we have successfully generated mice with deficient expression of seipin specifically in adrenal, testis, and ovary, steroidogenic tissues that accumulate CE-rich LDs under normal physiological conditions. The steroidogenic-specific seipin KO mice displayed a marked reduction in LD and cholesterol/CE accumulation, demonstrating the pivotal role of seipin in CE-rich LD accumulation/formation, in addition to its critical role in TAG-rich LDs. Moreover, the reduction in CE-rich LDs was associated with significant defects in steroid hormone production that could not be completely reversed by addition of exogenous cholesterol in the form of h-HDL_3_. We conclude that seipin has a heretofore unappreciated role in intracellular cholesterol trafficking.

## Data availability

All data are contained within the article.

## Supplemental data

This article contains supplemental data.

## Conflict of interest

The authors declare that they have no conflicts of interest with the contents of this article.
