# Supplementary material for: Mice deficient in ER protein seipin have reduced adrenal cholesteryl ester lipid droplet formation and utilization
Source: J Lipid Res. 2022 Nov 1;63(12):100309. doi: 10.1016/j.jlr.2022.100309 (PMC9703635; doi:10.1016/j.jlr.2022.100309)
Supplement: Supplemental Table 1 [file mmc1.docx]

| **Gene** | **Primer sequence** |
| --- | --- |
| 36B4-F | TTTGGGCATCACCACGAAAA |
| 36B4-R | GGACACCCTCCAGAAAGCGA |
| Plin1-F | CAG AGA GGT TAC AGC CCT GC |
| Plin1-R | CCC TCC CTT TGG TAG AGG AG |
| Plin2-F | AGCCAACGTCCGAGATTGTT |
| Plin2-R | CACATCCTTCGCCCCAGTT |
| HSL-F | GCT TGG TTC AAC TGG AGA GC |
| HSL-R | GGT AGA AGA GGG TCC ATG AGG |
| SR-B1-F | AAGTGGTCAACCCAAACGAG |
| SR-B1-R | ACGGTGTCGTTGTCATTGAA |
| LRP1-F | ACCACCAGCTACCTCATTGG |
| LRP1-R | CCTGGCCACACTAATGGTCT |
| LDLR-F | TCCTGGAGATGTGATGGACA |
| LDLR-R | GAGCCATCTAGGCAATCTCG |
| TMEM159-F | CCT AAG CAC CCA GAG GAG AG |
| TMEM159-R | ACA CAG TGA GCA GAG AAG CA |
| Star-F | CGGAGCAGAGTGGTGTCATC |
| Star-R | TGAGTTTAGTTTAGTCTTGGAGGGACTTC |
| Cyp21A1-F | CTTCTCTACTGGGGTGTGAGT |
| Cyp21A1-R | CGAGTTTTCTGTGCCCTTCA |
| 3ßHSD-F | TCCAGCTCAGTTGATGTTGC |
| 3ßHSD-R | TGCCTTCTCAGCCATCTTTT |
| α-SNAP-F | ACAGACATG GGCAG ATTCACA |
| α-SNAP-R | TGCAGATT GCTCGT AGTGG |
| SNAP25-F | AAATCGATACCCAGAATCGC |
| SNAP25-R | AACCACTTCCGAGCATCTTT |
| SNAP23-F | GGGCTCACCAGGTTACTGAT |
| SNAP23-R | ATGGTCTTAATTCCCGCATC |
| seipin-F | TCC TCT TTC CCA AGA ACG CT |
| seipin-R | GTG GGT CAC TGG AGC TAT GA |
| cyclophilin-A-F | AGCATACAGGTCCTGGCATCTTGT |
| cyclophilin-A-R | CAAAGACCACATGCTTGCCATCCA |

Supplemental Table S1. Primer sequences
